# A Study on Hot Stamping Formability of Continuous Glass Fiber Reinforced Thermoplastic Composites

**DOI:** 10.3390/polym14224935

**Published:** 2022-11-15

**Authors:** Feng Zhao, Wei Guo, Wei Li, Huajie Mao, Hongxu Yan, Jingwen Deng

**Affiliations:** 1 Hubei Key Laboratory of Advanced Technology for Automotive Components, Wuhan University of Technology, Wuhan 430070, China; 2 Hubei Collaborative Innovation Center for Automotive Components Technology, Wuhan University of Technology, Wuhan 430070, China; 3 School of Automotive Engineering, Wuhan University of Technology, Wuhan 430070, China; 4 Institute of Advanced Materials and Manufacturing Technology, Wuhan University of Technology, Wuhan 430070, China; 5 SAIC-GM-Wuling Automobile Co., Ltd., Liuzhou 545007, China; 6 School of Materials Science and Engineering, Wuhan University of Technology, Wuhan 430070, China

**Keywords:** thermoplastic resin matrix composites, hot stamping, part defect, thickness distribution, resin flow

## Abstract

In this study, hot stamping tests on continuous glass fiber (GF)-reinforced thermoplastic (PP) composites were conducted under different process parameters using a self-designed hemispherical hot stamping die with a heating system. The effects of parameters such as preheating temperature, stamping depth, and stamping speed on the formability of the fabricated parts were analyzed using optical microscopy and scanning electron microscopy (SEM). The test results show that the suitable stamping depth should be less than 15 mm, the stamping speed should be less than 150 mm/min, and the preheating temperature should be about 200 °C. From the edge of the formed parts to their pole area, a thin-thick-thin characteristic in thickness was observed. Under the same preheating temperature, the influence of stamping depth on the thickness variation of the formed parts was more significant than the stamping speed. The primary defects of the formed parts were cracking, wrinkling, delamination, and fiber exposure. Resin poverty often occurred in the defect area of the formed parts and increased with stamping depth and stamping speed.

## 1. Introduction

Since the 20th century, the explosive growth in the number of automobiles has brought great convenience to people while causing increasing pressure on energy and the environment. Reducing the weight of automobiles is significant for controlling energy consumption and reducing pollutant emissions [1]. Lightweighting technology, which replaces traditional steel materials with new lightweight and high-strength materials, is favored by the automotive industry [2].

Fiber-reinforced polymer (FRP) composites have received widespread attention among various new materials because of their light weight, high strength, corrosion resistance, fatigue resistance, and designability. They are widely used in aerospace structures, shipbuilding, and the automotive industry [3,4,5]. 

Due to the difference in length and shape, fibers can be divided into long fibers, short fibers, fiber cloth, fiber mat, etc. Compared with others, continuous fibers give full play to their reinforcing role in composites and significantly improve the strength of the components. Resins can be classified into thermosetting and thermoplastic resins due to their different properties. At present, continuous fiber-reinforced thermoset composites (FRTS) are widely used because of their thermal stability and chemical resistance. However, FRTS also has disadvantages such as low-temperature prepreg storage, long molding cycle, low strains-to-failure, and difficulty in re-forming [6,7]. Therefore, continuous fiber-reinforced thermoplastic (FRTP) composites, which are easy to store, have a short molding cycle, have high impact damage tolerance, and are easy to recycle [8,9], have replaced FRTS composites in many industries [10].

The conventional molding processes for continuous FRTP composites are compression molding, extrusion forming, tape winding, etc. [11,12,13]. Compared to the above methods, hot stamping is more suitable because of its advantages such as high productivity and adaptability to materials with complex shapes [3]. Research on the stamp forming of FRTP composites began in the 1980s [14]. The available relevant molding experiments have been mainly box-shaped experiments [15,16,17,18,19] and hemispherical experiments [20,21,22,23,24,25,26,27,28,29]. Zheng et al. [20] investigated the effect of stamping temperature and deformation rate on formability. Liu et al. [21] evaluated the effects of fiber reorientation and stamping-induced stresses during the molding process. Donadei et al. [16] investigated the effect of residual stress on the blank quality. Labanieh et al. [23] researched the connection between yarn slippage and forming defects.

The deformation mechanisms of hot stamping FRTP composites are resin penetration, transverse flow, interlayer sliding, and intra-layer shear [3,30,31,32]. Specifically, at the microscopic level, the deformation may be perceived as the contact mechanism between the resin and the fibers. At the mesoscopic level, the deformation can be considered as fiber bending and resin flow [30]. However, the deformation mechanism is not fully understood [23]. The forming temperature in most hot stamping experiments of FRTP composites is above the melting point (Tm) [3]. The fibers in FRTP composites are usually considered to be almost inextensible [22,24]. However, at high temperatures, the viscosity of the resin decreases, which affects the fiber movement, weakening the resin-fiber bond and intensifying the slippage between fibers [17,24,33]. The increased ductility of the matrix weakens the fiber/matrix interaction and reduces the protection of the fibers [20,34]. This leads to fiber damage, such as fiber leakage and breakage. The resin flow has a huge impact on the successful forming of composites. In particular, the resin flow phenomenon is prominent when using stacked materials [35]. For the hot stamping of FRTP composites, differences in resin flow due to different hot stamping parameters (depth, speed, temperature) are closely related to defects such as thickness mismatch, surface wrinkles, and fiber leakage of the molded part. However, relevant research has not been observed.

In past hot stamping experiments [20,21,22,23,24,25,26,27,28,29], scholars have preheated the workpieces outside the die and then quickly transferred them to the cold die for stamping and forming. A workpiece with a very small heat capacity cools significantly during the transfer process. Liu et al. [21] measured the cooling rate of the part in their experiments, up to 200 K/min. In their experiment, Tatsuno et al. [17] observed that the slower the cooling rate of the part, the higher its eventual strength. The cooling rate affects the degree of bonding between resin and fiber, which influences the mechanical properties of the final composite part [18,36,37]. In addition, the rapid cooling of the polymer matrix can prevent successful stamp forming [38]. When processing polymers for demanding applications (e.g., aerospace), the strength of the fiber-resin bond and the control of the microstructure is critical [15]. In this study, we designed a hemispherical hot stamping die. We heated the die and could control the temperature. The workpiece was kept at the same temperature during both preheating and stamping, which ensured the reliability of the experimental results. For small batches of FRTP composites with high processing performance requirements, our experimental design will provide a reference.

In the study, the hot stamping formability of glass fiber (GF)-reinforced polypropylene (PP) composites unidirectional laminates was investigated. First, the workpieces were obtained by hot stamping with different process parameters, including preheating temperature, stamping depth, and stamping speed. Then, the surface morphology of the workpiece was observed, and the thickness distribution of the workpiece was measured. Furthermore, the microstructure was observed using optical microscopy and scanning electron microscopy (SEM). Finally, the effects of hot stamping process parameters on the thickness distribution, fiber distribution, resin flowability, and defects of the workpiece were discussed in detail. The experimental results in this paper are useful for the selection of process parameters for hot stamping of GF/PP composites and have positive significance for expanding their use.

## 2. Experimental Materials and Methods

### 2.1. Raw Materials and Main Instruments

GF/PP laminate: 2-ply (0°/90°), with a thickness of about 0.58 mm, density of 1.5 ± 0.03 g/cm^3^, and fiber volume fraction of 60%, was obtained from Zhejiang Shenggang New Material Co., Ltd. (Taizhou, China). The properties of the grade E-glass fiber are shown in Table 1 [39], and the properties measured for the resin matrix PP are shown in Table 2.

### 2.2. Experimental Method

#### 2.2.1. Hot Stamping Molds

**Figure 1:**
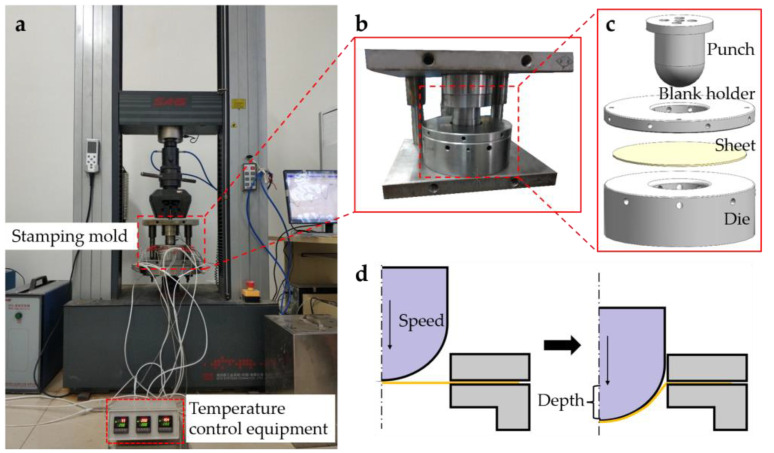
(**a**) The main equipment of the experiment; (**b**) the self-designed hemispherical hot stamping die; (**c**) the hot stamping die component; (**d**) the schematic diagram of hot stamping.

Figure 1a shows the main equipment of the experiment. Figure 1b,c show the self-designed hemispherical hot stamping die according to the experimental requirements. Figure 1d shows the schematic diagram of hot stamping. The stamping mold consisted of a punch, a blank holder, and a die. The punch was a hemisphere with a diameter of 60 mm; the diameter of the blank holder was 180 mm, and the gap between the concave die and the punch was 3 mm. The blank holder pressure was 0.15 Mpa. The friction coefficient between the composite material and the die parts was 0.2. To reduce the heat loss caused by the contact between the mold and the clamping parts, a heat insulation asbestos sheet was installed between the lower mold base and the mold support plate. The friction coefficient between the heat insulation asbestos sheet and the die was 0.3.

#### 2.2.2. Preparation Process and Experimental Protocol

The sheets required for the experiment were cut into 180 mm diameter circles. To prevent the molten resin from sticking to the mold, methyl silicone oil was used as a release agent. To avoid the accumulation of a small amount of resin remaining on the surface of the mold during the molding process, which would affect the release of the part, it was necessary to regularly grind and clean the surface contacted by the mold and the blank with fine sandpaper.

During the experiment, the temperature parameters were set on the temperature controller, and the stamping depth and speed were set on the testing machine. After the preheating reached the set temperature, it was maintained for 5 min. Then, the punch decreased to the set position at a certain speed and opened the mold to take part after cooling.

For the stamping temperature, 180 °C, 200 °C, and 220 °C were selected. Within the allowable test speed range of the universal testing machine, the gradient of the stamping speed was set to 50 mm/min. The stamping speed of 50 mm/min… 200 mm/min was selected, and the stamping depth ranged from 5 mm to 30 mm. The relevant experimental setups are shown in Table 3. Finally, at least three experiments were conducted for each condition.

#### 2.2.3. Testing and Characterization

**Figure 2:**
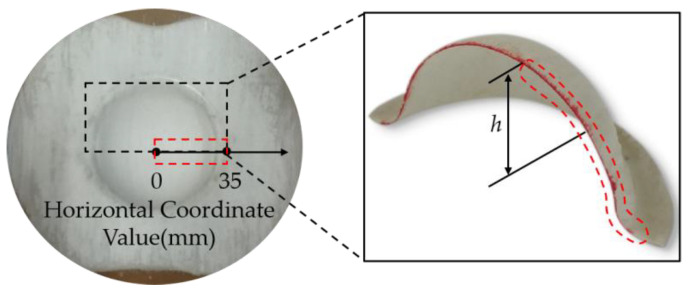
Thickness measurement area.

**Figure 3:**
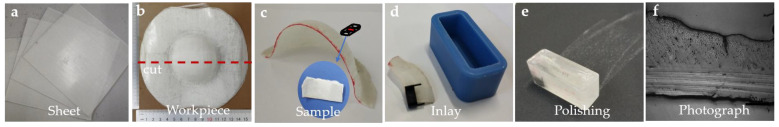
Sample preparation process: (**a**) Sheet; (**b**) workpiece; (**c**) sample; (**d**) inlay; (**e**) polishing; (**f**) photograph.

After the sample was prepared, thickness measurement and microscopic observation were performed. Due to the symmetry in the thickness of the part, half of the cross-section of the formed part was selected for thickness measurement, and the measurement area is shown in Figure 2. From the pole of the part (point 0) to the edge (point 35 mm), 7 points were taken for the thickness measurement on average. For microscopic observation, samples were taken sequentially with a diamond sander in the radius direction of the spherical area of the fabricated part, and the specimens were fixed using a slicing clamp and cold set in a soft silicone film. After the curing was completed, the specimens were ground and polished. Then, the specimens were observed and photographed under an optical microscope. SEM images were taken in the defective area of the parts to analyze the cause of the defects. The post-processing flow chart of the experiment is shown in Figure 3a–f.

## 3. Results and Discussion

### 3.1. Trend of Preheating Temperature

**Figure 4:**
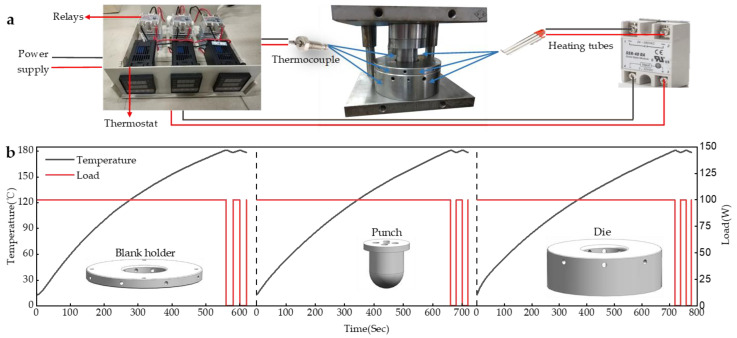
(**a**) The temperature control equipment; (**b**) preheating temperature variation curve: Blank holder/Punch/Die.

Figure 4a shows the temperature control equipment. The heating tubes were used to heat the mold. To accommodate the heating tubes, five, eight, and sixteen round holes were machined in the punch, blank holder, and die, respectively. Thermocouples were installed for real-time temperature monitoring, and the heating tubes were controlled by a temperature controller for constant temperature control.

Figure 4b shows the variation curve of the preheating temperature. During the preheating process, the blank holder reaches the preset temperature first, followed by the die and finally the punch. After the mold reaches the preset temperature, the temperature control device controls the heating tube for intermittent heating to ensure the temperature of the mold stays near the preset temperature. The maximum temperature error is 2 °C. It can be seen from the graph that the rate of temperature increase gradually became smaller as the temperature of the mold gradually increased. This is because when the heating power is constant, as the temperature of the mold increases, the temperature difference between the mold and the air increases, and the mold is more likely to exchange heat with the air, which leads to a slower heating rate.

### 3.2. Trend of Punching Pressure

**Figure 5:**
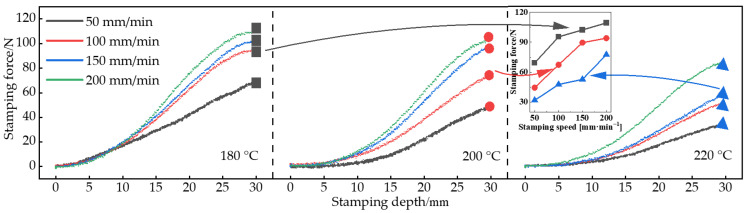
Stamping force variation curve: 180 °C/200 °C/220 °C.

Figure 5 shows the stamping force curves at different tamping speeds and stamping depths. From Figure 5, it can be seen that the stamping force increased with the increase of stamping speed and stamping depth during the hot stamping process of the part. This is because the resin matrix of the composite material is in a molten state after preheating, which reflects great viscosity in mechanical properties. The larger stamping speed and depth increase the flow rate of resin and produce a larger viscous force, which increases the deformation resistance of the composite. When the stamping depth is less than 10 mm, the stamping pressure curves overlap, while the slope of the stamping pressure curve increases significantly when the stamping depth exceeds 10 mm. As the stamping depth is small, the forces required to resist the deformation of the workpiece are smaller at different stamping speeds, resulting in a small difference in stamping force.

Further, the small graph in Figure 5 shows the variation curve of the maximum stamping force of the part. As shown in Figure 5, at the same stamping speed, as the preheating temperature increased, the viscosity of the resin in the composite decreased, and the stamping force required to stamp the same depth decreased. When the stamping speed was increased from 50 mm/min to 250 mm/min, the maximum stamping force required at the 180 °C, 200 °C, and 220 °C preheating temperature increased by 59.98%, 115.13%, and 158.97%, respectively.

### 3.3. Formability Analysis of Formed Parts

**Figure 6:**
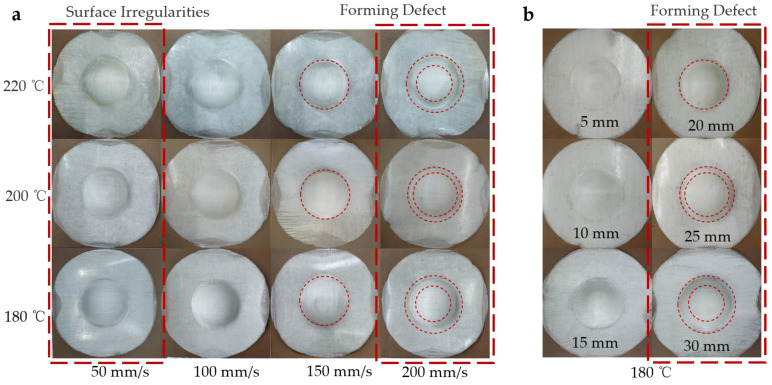
The macroscopic shape of the formed part: (**a**) Shape of the part at different stamping speeds and preheating temperatures; (**b**) shape of parts with different stamping depths at 180 °C.

In the hot stamping process of continuous GF/PP laminates, the preheating temperature, stamping speed, and stamping depth are closely related to the macroscopic morphology of the parts. Figure 6a shows the shape of the part at different stamping speeds and preheating temperatures. Figure 6b shows the shape of the parts at different stamping depths. Since there were too many experimental molded parts, only some representative parts are shown for analysis and illustration.

As shown in Figure 6a, when the preheating temperature was 180 °C, the surface quality of the part was poor and the surface was rough. With the increase of the stamping speed, a particular wrinkling phenomenon appeared from the vicinity of the waist to the edge of the part. Significantly, when the stamping speed exceeded 150 mm/min, the wrinkling phenomenon became obvious. When the preheating temperature was 200 °C, the surface quality of the formed parts was good. The wrinkling phenomenon between the waist and the edge of the part was more obvious only when the stamping speed reached 200 mm/min. When the preheating temperature was 220 °C, the surface of the part was smooth, and the surface quality of the pole area of the part was further improved. However, when the stamping speed was too high, the workpiece easily produced defects such as composite material delamination and fiber fracture. The main reason is that the increase in preheating temperature softens the matrix, increases the ductility of the matrix, and attenuates the interaction between the fiber and the matrix, which easily leads to the delamination of the composite material and the bare fiber leakage. With the increase of stamping speed, the relative motion of fiber and resin is aggravated, which is more likely to lead to workpiece defects. In general, when the stamping speed is less than 100 mm/s, the surface quality of the parts is poor. When the stamping speed is greater than 150 mm/s, the parts are prone to forming defects. When the preheating temperature is 200 °C, the forming quality of the workpiece is better. Forming defects occur between the waist and the edge of the workpiece; the main defects are cracking, wrinkling, delamination, and so on, and the defects often occur together. Related experiments have also confirmed that higher preheating temperatures are often beneficial and can improve the quality of the workpiece [16,40,41]. The increase in stamping speed often leads to a decrease in formability [20].

Figure 6b shows the macroscopic morphology of the workpiece when the preheating temperature was 180 °C and the stamping speed was 200 mm/min. The forming quality of the parts was better when the stamping depth was less than 15 mm, and with the increase of the stamping depth, the phenomenon of pulling cracks and bare fiber leakage at the edges of the parts intensified, and the area of the ring defects increased. The main reason is that as the stamping depth increases, the deformation of the formed workpiece increases, and the area involved in the deformation also increases internally, which eventually leads to an increase in the defects of the formed workpiece.

### 3.4. Forming Defects of Formed Parts

**Figure 7:**
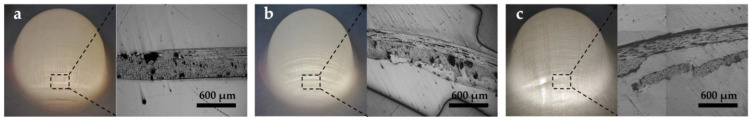
Forming defects of the parts: (**a**) The fiber fracture of the parts; (**b**) the wrinkle situation of the parts; (**c**) the delamination of the parts.

The forming defects of the parts are shown in Figure 7, whereas Figure 7a–c show fiber breakage, wrinkling, and delamination of the workpiece, respectively.

Figure 7a shows the fiber fracture of the parts. The fractured fibers were located in the inner layer of the composite. This is because the deformation rate of the workpiece increases when the moving speed of the punch is high. At higher stamping speeds, the lack of resin isolation of the interlayer fibers leads to enhanced interlayer shearing of the fibers. Since the shear resistance of glass fibers is weaker than the tensile resistance, the inner layer fibers are prone to break under the action of shear force. Moreover, when the stamping temperature is low, the toughness of the matrix is poor, and the interaction between the matrix and fiber is strong. The large deformation of composite materials can easily lead to fiber fracture.

Figure 7b shows the wrinkle situation of parts. The essential cause of wrinkles was the local stacking of fibers. This defect was mainly found in the area between the waist and the edge of the part. During the hot stamping process, the punch is in direct contact with the inner layer of the composite material. When the stamping speed is too fast or the stamping depth is too large, the deformation produced by in-plane extrusion increases. However, the deformation of the composite is not uniform due to the uneven fiber distribution and resin content between the layers in the composite. The outer layer of fibers moves and bends under less tension, which tends to produce localized fiber stacking and eventually evolves into a wrinkling phenomenon at the end of stamping.

Figure 7c shows the delamination of parts, which also occurred near the waist area of the part. The main reason is that the increase in preheating temperature softens the matrix, increases the ductility of the matrix, and weakens the interaction between the fibers and the matrix. When the deformation of the composite is large, relative motion tends to occur between the composites, resulting in delamination defects.

**Figure 8:**
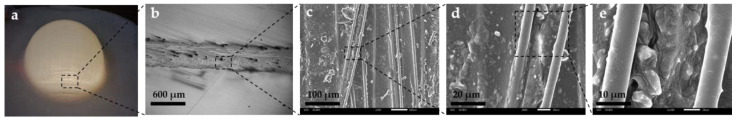
SEM observation of the forming defect area: (**a**) Macrophotograph; (**b**) 600 µm; (**c**) 100 µm; (**d**) 20 µm; (**e**) 10 µm.

Figure 8 shows the SEM observation of the forming defect area. As can be seen from the figure, the fibers separated from the resin were morphologically intact, with a moderate amount of resin residue on the surface. The fibers can still maintain a relatively uniform arrangement in most of the exposed areas of the fibers mentioned above. It can be judged that the resin was separated from the fibers in the molten state, and the resin was easily lost in the blank due to the low viscosity and good flowability of the resin above the melting point. In this way, the protective effect of the resin on the fiber was weakened, and eventually, the composite material was damaged.

### 3.5. Thickness Distribution of Formed Parts

**Figure 9:**
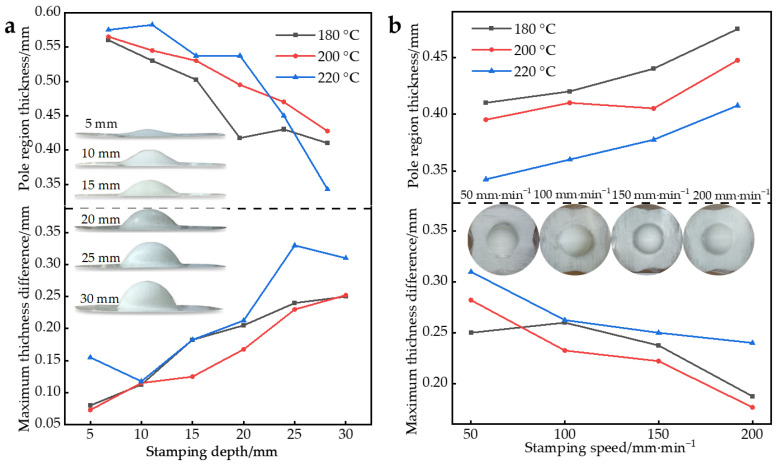
(**a**) Variation curve of pole thickness and maximum thickness difference within the parts under different preheating temperatures and stamping depth; (**b**) variation curve of pole thickness and maximum thickness difference within the parts under different preheating temperatures and stamping speed.

Further, the thickness variation of the molded parts was studied. Figure 9a shows the maximum thickness difference within the part and the pole thickness at different preheating temperatures and stamping depths at a speed of 100 mm/min. Figure 9b shows the maximum thickness difference within the part and the curve of the pole thickness at different preheating temperatures and stamping speeds with a stamping depth of 30 mm. The thickness of the part is the average of three measurements.

As shown in Figure 9a, with the increase of the stamping depth, the pole thickness of the part gradually decreased, and the thickness difference of the part gradually increased. With the increase of the stamping depth, the part reflected a poor uniformity in thickness. This is because the resin flow is aggravated with the increase of stamping depth, and the inhomogeneity of resin flow leads to the difference in forming quality.

As shown in Figure 9b, with the increase in stamping speed, the pole thickness of the part gradually increased, and the thickness difference of the part gradually decreased. The increase in stamping speed showed a better uniformity in the thickness of the part. This is because when the stamping speed is fast, the deformable time is shorter than the resin relaxation time, and the thickness change is not apparent. When the stamping speed is slow, the deformable time is obviously longer than the relaxation time and the thickness changes.

Overall, as the preheating temperature increases, the fluidity of the resin increases, resulting in a thinner thickness in the pole region. However, when the preheating temperature is too high, it will lead to poor uniformity of the formed parts. In this experiment, the most suitable preheating temperature was 200 °C.

**Figure 10:**
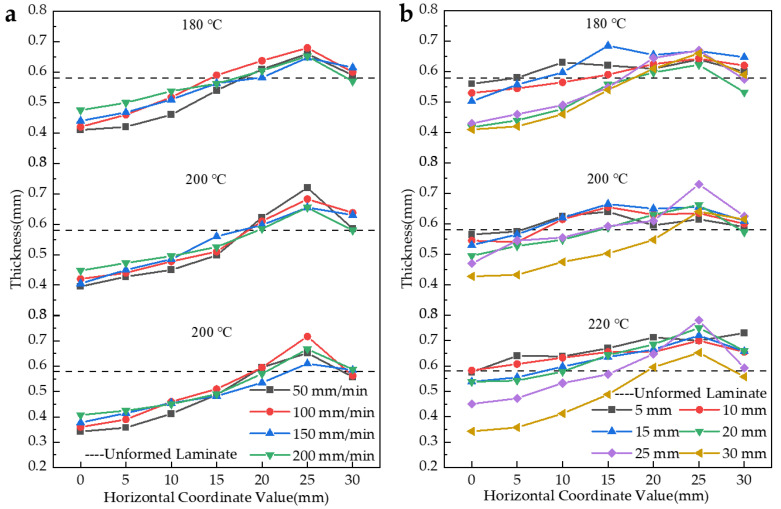
Thickness distribution of parts at different preheating temperatures: (**a**) Stamping depth of 30 mm at different stamping speeds; (**b**) different stamping depths at a speed of 100 mm/min.

Further, the specifics of the thickness pickup point distribution of the parts in Figure 9 are discussed, as shown in Figure 10.

From Figure 10, in general, the thickness showed a thin-thick-thin characteristic in each formed part. The thinnest point of the part was at the pole, and the thickest point appeared at the 25 mm point. When stamping began, the hemispherical punch and concave die that were heated came into contact with the workpiece. The high temperature in the contact zone made the resin highly elastic and even viscous flowing, making the workpiece easy to thin, while the temperature in the uncontacted zone was lower and the thickness change was not obvious during stamping. In addition, the presence of the blank holder prevented the flow of resin in part, resulting in the accumulation of resin in the waist region. Another important reason is that the defects of the formed workpiece, such as fiber breakage, wrinkling, delamination, etc., led to uneven thickness of the workpiece.

In general, the trend of thickness variation was similar for each formed part. However, comparing Figure 10a,b, it is evident that the curves in Figure 10a overlap each other and the curves in Figure 10b are distributed over a larger area. This indicates that the effect of stamping depth on the thickness variation of the formed parts was significantly greater than stamping speed at the same preheating temperature.

### 3.6. Mesoscopic Structure of Formed Parts

**Figure 11:**
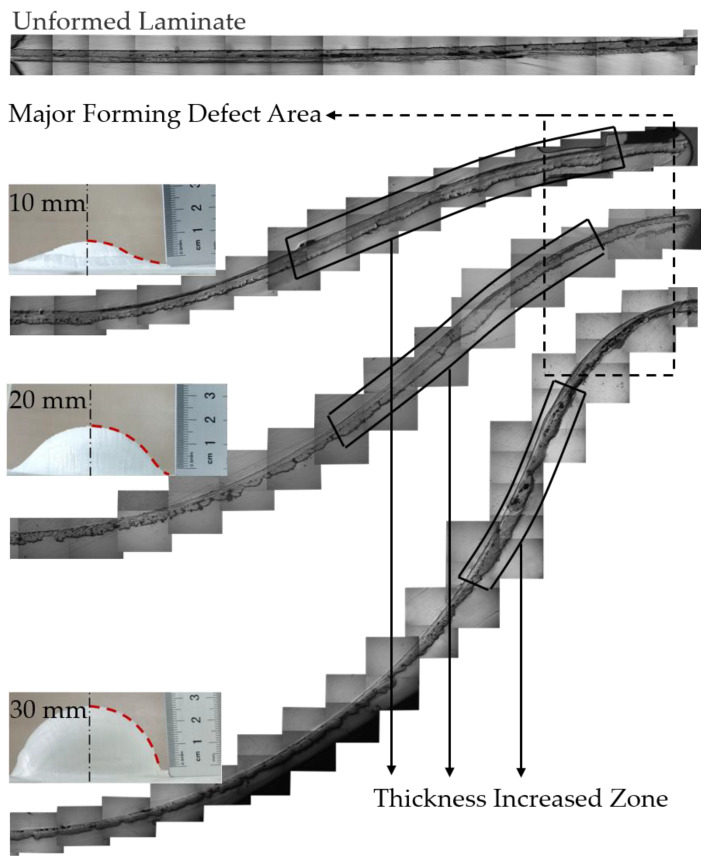
Microscope images of the formed parts.

During the high-temperature stamping process, the resin flow inside the part significantly impacted its forming quality. After the above analysis, the stamping depth had a more noticeable effect on the formability of the molded parts. Therefore, for a more comprehensive quality assessment of the fabricated parts, the mesoscopic morphology of the formed parts with stamping depths of 10 mm, 20 mm, and 30 mm was analyzed at a preheating of 200 °C and stamping speed of 100 mm/min. The mesoscopic morphology of the 1/4 section of the manufactured part was observed, and the results are shown in Figure 11.

When the depth was below 10 mm, the resin flowed less during the molding process due to the limited deformation of the part. Therefore, the cross-sectional shape of the part was more regular, the distribution of resin and fiber was more uniform, and the probability of quality defects in the part was low. The resin flow rate increased during the forming of 20 mm depth parts, and the resin content in the area near the poles decreased, which reduced the coating effect of the resin on the fibers and reduced the surface quality of the parts. However, the resin content in the area near the waist of the part increased significantly. For a 30 mm depth part, the resin was mainly distributed between the waist and the edge, where wrinkling occurred due to the local accumulation of isotropic fibers. Overall, after forming, the inside of the part had a flat surface due to the fit of the mold, and the outside surface of the part was uneven. This was due to the close fit between the inner surface of the part and the punch, which regulated the flow behavior of the resin. Whereas the outer surface of the part was not in contact with the die, the resin was easily lost under the traction of the punch. This eventually led to defects such as fiber exposure and made the outer surface molding quality rougher. The thickness of the formed part was not uniform, and the thickening area decreased with the increase of the stamping depth. The thickening area was concentrated near the waist of the part, forming a resin aggregation phenomenon. The defective area of the formed part was mainly concentrated in the annular area between the waist and the edge. The traction force generated by the die in this area reduced the ability of the composite to retain the resin, thus making the part prone to forming defects. For the composite material hot stamping process, the complete contact between the part and the die can improve the ability to retain the resin for the composite material and improve the forming quality.

## 4. Conclusions

In this paper, the forming properties of continuous GF/PP laminates were investigated through hemispherical hot stamping experiments, and the main conclusions were obtained as follows.

(1) In the hot stamping process, the stamping pressure increased with the increase of stamping speed and stamping depth and decreased with the increase of preheating temperature.
(2) Forming defects can be effectively avoided when the stamping depth is less than 15 mm and the stamping speed is less than 150 mm/min. The preheating temperature should be set at around 200 °C. Under the same preheating temperature, the influence of stamping depth on the thickness variation of the formed parts was more significant than the stamping speed.
(3) The overall thickness of the part showed thin-thick-thin characteristics. The thinnest point of the part was at the pole, and the thickest point of the part appeared at 25 mm from the pole. With the increase of the stamping depth, the part reflected a poor uniformity in thickness. With the increase in stamping speed, the uniformity of the formed parts was better.
(4) Forming defect areas were often accompanied by resin loss and aggregation. Improving the composite’s ability to retain resin can avoid forming defects.

## Figures and Tables

**Table 1:** Properties of the E-glass fiber.

Parameters	Value
*E_1_*, *E_2_*, *E_3_* (Gpa)	70.5
*G_12_*, *G_13_*, *G_23_* (Gpa)	29.375
*v_12_*, *v_13_*, *v_23_*	0.2
*ρ* (kg/m^3^)	2570
*CTE_1_*, *CTE_2_*, *CTE_3_* (με/°C)	5.4
*k_1_*, *k_2_*, *k_3_* (W·(m·K)^−1^)	1.3
*C* (kJ·(kg·K)^−1^)	0.67

**Table 2:** Properties of the resin matrix PP.

Parameters	Value
$v^{0}$	0.415
$v^{\infty }$	0.497
*ρ* (kg/m^3^)	910
*CTE* (με/°C)	130
*k* (W·(m·K)^−1^)	2.53
*C* (kJ·(kg·K)^−1^)	0.1889
*M_w_* (10^4^)	26.49
*M_n_* (10^4^)	3.35
*X_c_* (%)	38.6
*T_g_* (°C)	−15
*T_m_* (°C)	168.46

**Table 3:** Hot stamping experimental setup.

Parameter	Preheating Temperature/°C	Velocity/mm·min^−1^	Depth/mm
value	180, 200, 220	50, 100, 150, 200	5, 10, 15, 20, 25, 30

## Data Availability

The data presented in this study are available on request from the corresponding author.
